# Rethinking and Co-Design of Beloved Board Games for People With Mild Cognitive Impairment and Their Care Partners

**DOI:** 10.1093/geroni/igaa057.1836

**Published:** 2020-12-16

**Authors:** Chantal Kerssens, Maribeth Gandy, Kara Cohen, Laura Levy, Cecile Janssens, Tracy Mitzner, Molly Perkins, Suzette Binford

**Affiliations:** 1 Georgia Institute of Technology, Atlanta, Georgia, United States; 2 Emory University, Atlanta, Georgia, United States; 3 Emory Brain Health Center, Atlanta, Georgia, United States

## Abstract

Mild cognitive impairment (MCI) affects millions of older Americans and progression to dementia is common. Although people with MCI may experience impairments, they are often highly verbal, able, and eager to uphold beloved routines. Moreover, many seek opportunities to stay active, physically and mentally, to support their brain health. Some forms of cognitive training and social engagement potentially delay the onset and progression of disease, including dementia. This 12-month project used mixed methods to co-design and test an accessible version of well-known board games for people with MCI and a care partner without MCI. The overall goal was to foster a meaningful, joyous, social activity for players with differing capabilities using adapted game mechanics to create a compelling experience for both players. Coping strategies of care partners were studied to learn ways to foster positive interactions. Findings inform recommendations for game design and clinical interventions. Part of a symposium sponsored by Technology and Aging Interest Group.

